# Effect of sodium bicarbonate infusion on hospital mortality in acute kidney injury patients with metabolic acidosis

**DOI:** 10.3389/fmed.2023.1268252

**Published:** 2023-10-12

**Authors:** Yunting Wang, Ling Chen, Guangfeng Guo, Youyuan Gao, Hua Gan

**Affiliations:** ^1^Department of Nephrology, The First Affiliated Hospital of Chongqing Medical University, Chongqing, China; ^2^The Chongqing Key Laboratory of Translational Medicine in Major Metabolic Diseases, The First Affiliated Hospital of Chongqing Medical University, Chongqing, China

**Keywords:** AKI, metabolism acidosis, sodium bicarbonate, hospital mortality, MIMIC

## Abstract

**Background:**

Physicians usually consider that sodium bicarbonate (SB) infusion can be used for metabolic acidosis; however, there is little evidence available to assess its effect on hospital mortality in large AKI cohorts. Here, we investigated the effect of SB infusion in patients with AKI complicated by metabolic acidosis.

**Method:**

Patients with AKI complicated by metabolic acidosis were screened from the MIMIC-IV database. A propensity score analysis (PSA) was used to decrease baseline differences in the probability of receiving SB. The marginal structural Cox model (MSCM) was employed to adjust for both baseline and time-varying confounding factors.

**Results:**

A total of 1853 patients with AKI complicated with metabolic acidosis were included in our study. A total of 390 pairs of patients were divided into an SB infusion group and a non-SB infusion group. The SB infusion group had more serious and worse laboratory indicators, including lower pH [7.19 (0.11) vs. 7.26 (0.07)] and bicarbonate concentration (BC) [12.36 (4.26) vs. 15.96 (3.25) mmol/l]. While there was no significant effect on overall hospital mortality in AKI patients complicated with metabolic acidosis (*p* = 0.056), SB infusion was observed to have beneficial correlation on hospital mortality in patients with high AG acidosis (AG > 18 mmol/L) (*p* = 0.012). Similar results were replicated with the MSCM.

**Conclusion:**

We found that SB infusion in AKI patients with metabolic acidosis is not beneficial for hospital mortality. However, SB infusion for AKI patients and high AG metabolic acidosis significantly improved hospital mortality. Further larger randomized controlled trials are needed to confirm these results.

## Introduction

Over the past few decades, the incidence of acute kidney injury (AKI) has increased, and it is a common complication in patients admitted to intensive care units (ICUs), and is closely associated with adverse outcomes such as prolonged ICU and hospital stay, development of chronic kidney disease (CKD), and increased risk of short-and long-term mortality (1–3). In ICUs, the probability of AKI leading to death is up to 50% (4).

AKI is not a single disease, but a syndrome consisting of multiple clinical conditions. The prognosis of AKI depends on the underlying disease, the severity and duration of renal impairment, the patient’s renal baseline condition, and the treatment status (5). Fluids are one of the most commonly used therapies for critically ill patients and represent the cornerstone of hemodynamic management in ICUs. The use of fluids to optimize hemodynamics during the perioperative and early stages of sepsis has been shown to improve patient outcomes (6). On the other hand, liberal administration of fluids may lead to a positive fluid balance, and recent observational studies have demonstrated an association between positive fluid balance, renal non-recovery, and mortality in children and adults with AKI (7).

Acidosis is the most common acid–base disturbance seen in AKI, and recent studies have shown that AKI with metabolic acidosis potentially indicates a more severe course and worse outcome, including high mortality and increased length of stay in the hospital and ICU (8). On the one hand, AKI can cause metabolic acidosis; on the other hand, accumulating evidence identifies metabolic acidosis not only as a consequence of, but also as a contributor to the progression of kidney dysfunction (9). Anion gap (AG), an indicator that reflects the unmeasured anion concentration, is applied to detect and evaluate the presence and severity of metabolic acidosis. While AKI has been investigated widely for hospital mortality, AKI in association with metabolic acidosis has been less well studied.

Sodium bicarbonate (SB) infusion is mostly administered to treat severe metabolic acidosis. However, it is controversial whether SB should be used and which dosage should be used in clinical practice (10, 11). A retrospective observational study revealed that SB infusion may reduce ICU mortality in septic patients who presented with AKI stage 2 or 3 (12). Therefore, we hypothesized that early sodium bicarbonate infusion may be able to correct acidosis and halt its progression, thereby mitigating the risk of additional renal damage and resulting in lower mortality in patients with AKI.

## Materials and methods

### Source of data

This study was conducted by collecting data from a real-world and publicly available clinical database named the Multiparameter Intelligent Monitoring in Intensive Care Database IV (MIMIC IV), which included more than 60,000 ICU patients treated at Beth Israel Deaconess Medical Center (Boston, MA) from 2008 to 2019, as previously described (13). One author, (Yunting Wang), had access to the database and was responsible for data extraction (certification ID: 41733658). Because the database here had already been approved by the institutional review board (IRB) of the Massachusetts Institute of Technology (MIT), exemption from additional institutional IRB approval was allowed (14).

### Participants and variables

The inclusion criteria were patients (1) diagnosed with AKI; (2) with metabolic acidosis with pH < 7.3 and bicarbonate <20 mmoL/L; and (3) who did not have respiratory acidosis (PaCO2 < 50 mmHg) (12). The minimum values were used for BC and pH, and the maximum value was used for PaCO2 within 48 h after ICU entry. In this study, all adult patients met the criteria for AKI within 48 h after ICU admission according to the Kidney Disease Improving Global Outcomes (KDIGO) clinical practice guidelines (15), and both urine output and creatinine were used to define AKI stages. If patients were admitted multiple times, only the first stay was analysed. Patients aged <18 years old and those who were discharged or died within 48 h after ICU admission were excluded.

We extracted clinical data within 24 h after admission to the ICU, including age, sex, mechanical ventilation, and sequential organ failure assessment (SOFA) score. In addition, laboratory data, including chloride, potassium, sodium, lactate, creatinine, and blood urea nitrogen (BUN), were included. If a variable was measured more than once in the first 24 h, the value associated with the greatest severity of illness was used. Because missing data may create bias, variables with >70% missing values were excluded from further analysis. Other variables with a lesser degree of missing values were analysed using the multiple imputation method (16).

The primary outcome was patient mortality in the hospital and ICU. Both were identified as the outcomes of patient survival on discharge from the hospital and ICU.

### Statistical analysis

According to whether patients were infused with SB 48 h after ICU entry, we divided the investigated cohort into two groups: the intervention (SB) group and the control (non-SB) group. Continuous variables are expressed as the mean and standard deviation (SD) and interquartile ranges (IQR). The chi-square test or Fisher’s exact test was used to test comparisons between the groups for categorical variables, whereas the t test or Mann–Whitney U test was used for comparisons of continuous variables, as appropriate. Categorical variables are presented as numbers and proportions (%). All statistical analyses were performed by the R package (version 3.6.3). *p* values <0.05 were considered statistically significant for all analyses.

We used propensity score analysis (PSA) to account for the baseline differences in the probability of receiving SB or not within 48 h after ICU entry so that covariate imbalance between the SB and no SB groups was minimized (17). The standardized mean difference (SMD) was calculated and was significantly smaller after group matching. A Cox regression model was used to adjust for residual imbalance by including parameters with statistically significant *p* value and based on clinical expertise advice of any potential confounders.

Marginal structural Cox models (MSCM) provide the time-dependent relation between a time-varying variable (SB treatment) and a survival outcome during hospitalization after ICU admission. Age, sex, mechanical ventilation, total fluid input, vasopressor, BUN, creatinine, urine output, SOFA score, anion gap (AG), and sodium and potassium levels were obtained in the first 24 h after ICU admission as potential baseline variables. BC, PaCO2, lactate and PH were used as time-varying variables in the entire ICU period. Inverse probability weighting estimation in R and the “ipw” package. Were performed on MSCM parameters to correct confounding and selection bias formed by informative censoring.

## Results

**Figure 1:**
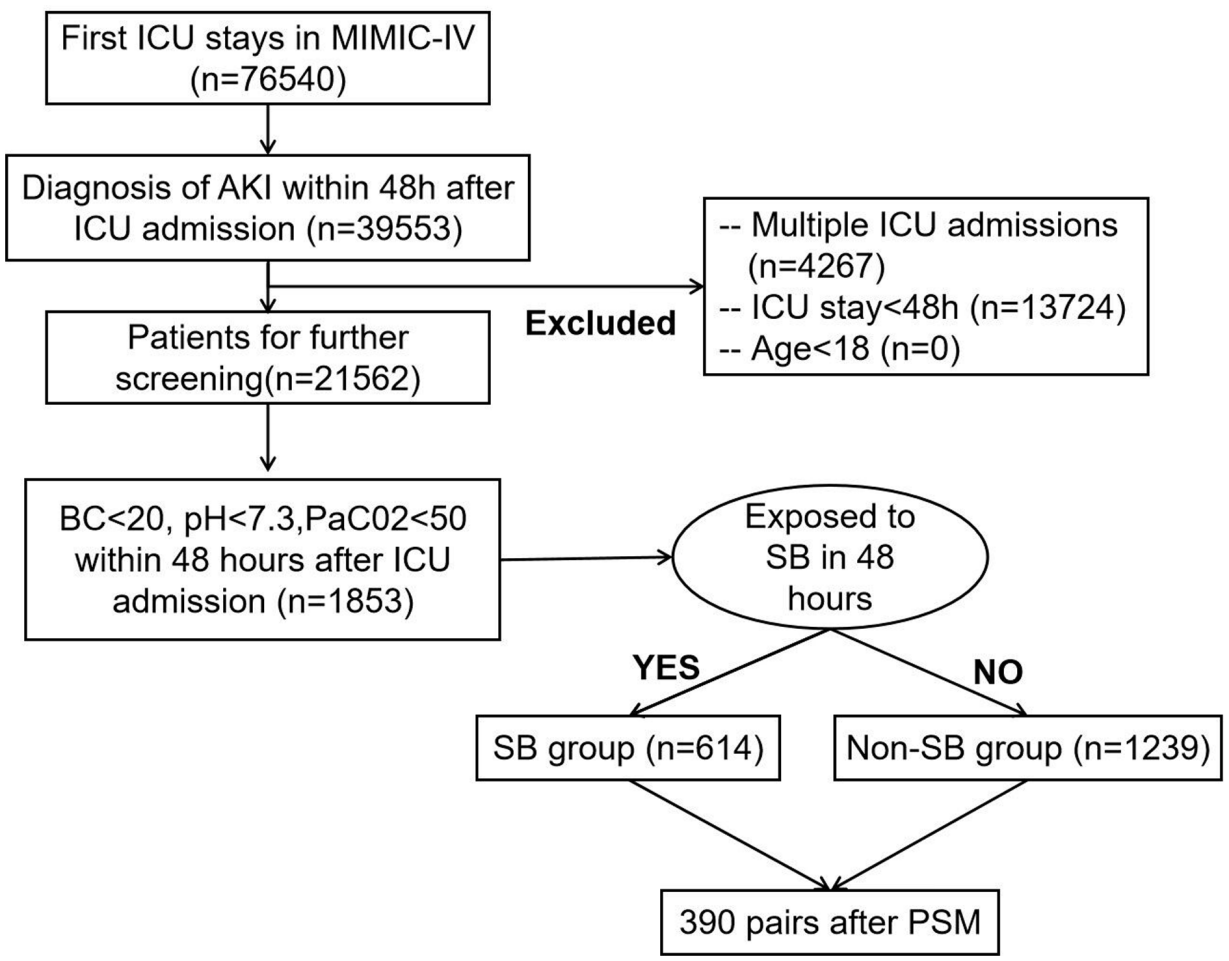
Study cohort selection based on the inclusion and exclusion criteria.

We identified AKI adult patients according to the KDIGO criteria, and after including only adult patients’ first ICU admissions, 1853 AKI patients with metabolic acidosis 48 h after ICU admission from the MIMIC-IV database were included in our study. Among the study cohort, 614 patients received SB treatment, and 1,239 patients did not receive SB treatment in the first 48 h after entering the ICU department (Figure 1).

The baseline characteristics of the SB treatment group and non-SB treatment group before and after PSA matching are shown in Table 1. The SB treatment group had a more serious situation than the non-SB group. The SB treatment group had significantly higher severity scores on admission [SOFA 9.0 (7.0, 11.0) vs. 7.0 (5.0, 9.0)]. A larger proportion of the SB patients received vasopressors (36.5% vs. 17.1%) during the first 24 h of their ICU stay, but there were few differences in mechanical ventilation. In the first 24 h of admission, PH [7.19 (0.11) vs. 7.26 (0.07)], BC [12.36 (4.26) vs. 15.96 (3.25) mmol/L] and urine output [684.00 (264.25, 1399.25) vs. 1000.00 (561.00, 1667.00) mL] were significantly lower in the SB group. On the first day, fluid input [3825.00 (2081.25, 6257.50) vs. 2300.00 (1100.50, 3922.50) mL] and anion gap [24.23 (7.79) vs. 19.89 (5.82)] was significantly higher in the SB treatment group. Renal function, including creatinine [2.60 (1.60, 4.10) vs. 1.60 (1.10, 2.70) mg/dL] and BUN [42.00 (26.00, 67.00) vs. 32.00 (20.00, 51.50) mmol/L], was more serious in the SB treatment group.

**Table 1:** Comparison of the basic covariates between the original cohort and the adjusted cohort.

	Original cohort				Matched cohort			
	Non-SB	SB	SMD	*p*	Non-SB	SB	SMD	*p*
*n*	1,239	614			390	390		
Age	66.66 (15.91)	64.62 (15.61)	0.130	0.009	66.55 (15.52)	65.51 (15.77)	0.067	0.351
Gender (Female)	559 (45.1)	299 (48.7)	0.072	0.151	191 (49.0)	185 (47.4)	0.031	0.72
SOFA score	7.0 (5.0,9.0)	9.0 (7.0,11.0)	0.636	<0.001	8.00 [6.00, 10.00]	8.00 [6.00, 10.00]	0.032	0.596
Mechanical ventilation	1,014 (81.8)	482 (78.5)	0.084	0.091	81 (20.8)	81 (20.8)	<0.001	1
Vasopressor	212 (17.1)	224 (36.5)	0.448	<0.001	96 (24.6)	96 (24.6)	<0.001	1
Urine output	1000.00 (561.00, 1667.00)	684.00 (264.25, 1399.25)	0.134	<0.001	850.50 [420.00, 1436.75]	819.50 [335.00, 1543.75]	0.03	0.796
AKI stage, *n* (%)			0.466	<0.001			0.061	0.707
1	243 (19.6)	72 (11.7)			50 (12.8)	54 (13.8)		
2	514 (41.5)	164 (26.7)			139 (35.6)	128 (32.8)		
3	482 (38.9)	378 (61.6)			201 (51.5)	208 (53.3)		
Fluid input	2300.00 (1100.50, 3922.50)	3825.00 (2081.25, 6257.50)	0.59	<0.001	2829.00 [1484.25, 4748.25]	2999.50 [1735.75, 5037.50]	0.015	0.363
Lactate	3.86 (2.78)	5.93 (4.72)	0.535	<0.001	4.53 (3.34)	4.63 (3.74)	0.027	0.708
PH	7.26 (0.07)	7.19 (0.11)	0.717	<0.001	7.23 (0.09)	7.23 (0.09)	0.02	0.781
PCO2	41.56 (5.78)	38.98 (7.69)	0.378	<0.001	39.97 (6.63)	40.25 (6.88)	0.04	0.574
Bicarbonate	15.96 (3.25)	12.36 (4.26)	0.949	<0.001	14.11 (3.64)	14.12 (3.81)	0.004	0.951
BUN	32.00 (20.00,51.50)	42.00 (26.00,67.00)	0.382	<0.001	38.00 [24.25, 64.00]	39.00 [25.00, 63.00]	0.01	0.743
Creatinine	1.60 (1.10,2.70)	2.60 (1.60,4.10)	0.284	<0.001	2.00 [1.40, 3.30]	2.40 [1.50, 3.80]	0.018	0.014
Anion gap	19.89 (5.82)	24.23 (7.79)	0.631	<0.001	21.76 (6.54)	22.28 (6.92)	0.077	0.283

After propensity score analysis, we matched 390 pairs of SB treatment and non-SB treatment patients in subsequent studies. The imbalance of variables was reduced, and all variables had an SMD value <0.1. In our study, SB infusion was not associated with improved mortality in all AKI patients after PSA matching (*p* = 0.056). However, SB infusion is associated with beneficial mortality in AKI patients with high AG metabolic acidosis (*p* = 0.012), as shown in Table 2. Our further study found that the use of SB was an important factor correlating with the mortality outcome in AKI patients with high AG acidosis (*p* = 0.021), as shown in Table 3.

**Table 2:** Relation of SB use and hospital mortality in the overall and subgroups using Cox proportional hazard model analysis.

Groups	HR	Lower.95	Upper.95	*p*
Overall population	0.85	0.71	1.00	0.056
AKI stage 1 (*n* = 104)	0.71	0.44	1.14	0.159
AKI stage 2 (*n* = 267)	1.05	0.80	1.39	0.725
AKI stage 3 (*n* = 409)	0.79	0.61	1.02	0.065
AG>18 (*n* = 529)	0.76	0.61	0.94	0.012

**Table 3:** Cox regression model after propensity score matching in AKI patients with AG>18.

Variables	HR	Lower.95	Upper.95	*p*
Use of SB	0.77	0.62	0.96	0.021
Age	1.01	1.00	1.02	0.021
PH minimum	0.48	0.14	1.61	0.236
PaCO2 max	0.99	0.98	1.01	0.522
BC min	0.96	0.93	1.00	0.074
Ventilation	1.07	0.81	1.40	0.649
Urine output	1.00	1.00	1.00	0.372
BUN	1.00	1.00	1.00	0.439
Creatinine	1.01	0.99	1.03	0.580
Lactate max	0.95	0.92	0.98	0.002
Total fluid input	1.00	1.00	1.00	0.766

**Figure 2:**
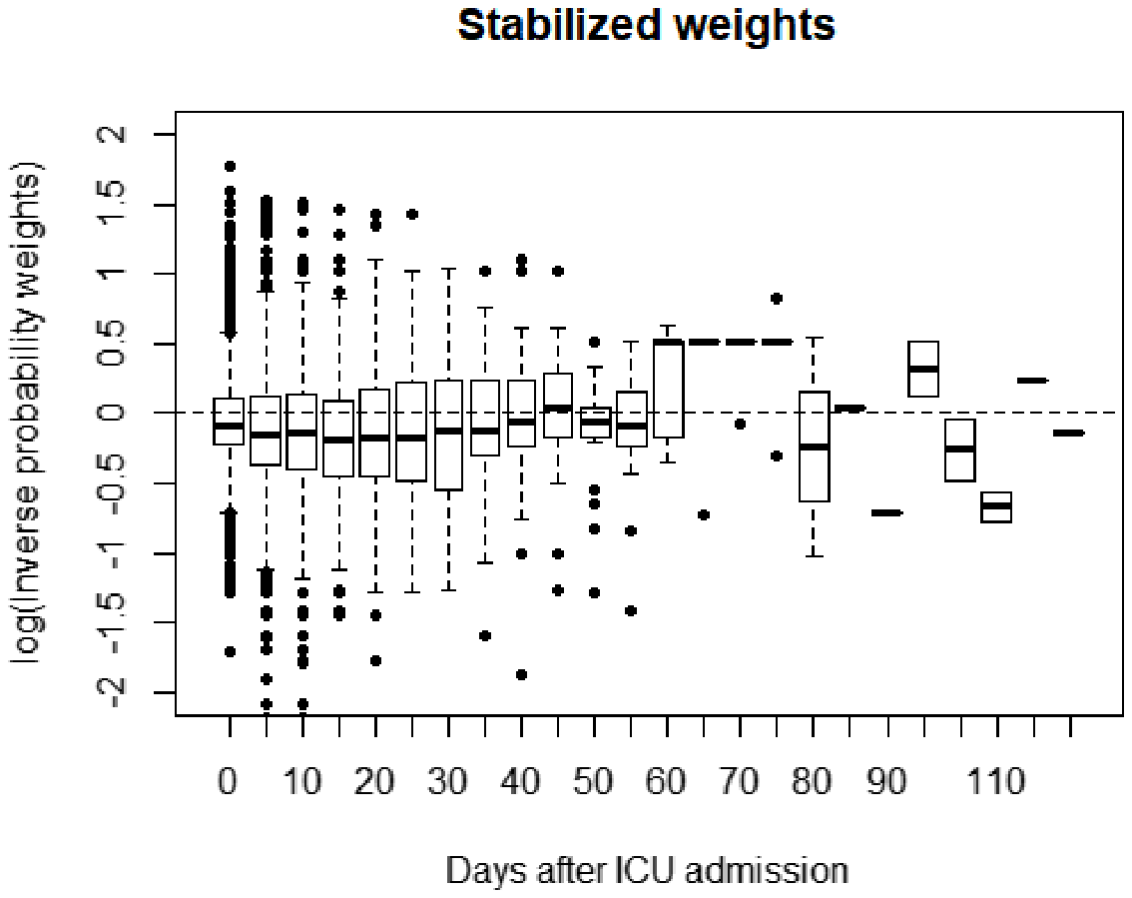
Weight distribution plot for the inverse probability weights that were used to adjust for confounding.

In our study, variables could be estimated using inverse probability weighting (IPW) to correct both for confounding and for forms of selection bias (Figure 2). PH, BC and lactate were the most important predicting variables of SB use; among them, pH ((for each 0.1) increase, *p* < 0.001), BC (for each 5 mmoL/L increase, *p* < 0.001) and lactate (for each 1 increase, *p* = 0.002), as time-varying variables, all have significance (Table 4). Our results revealed that SB treatment was not associated with improving hospital mortality in the overall cohort of patients (*p* = 0.179). MSCM showed that AKI stage 1 to 3 patients had a less significant relationship between SB use and mortality (AKI phase 1, *p* = 0.126; AKI phase 2, *p* = 0.131; AKI phase 3, *p* = 0.106). SB use still closely correlated with the improving outcome of the AKI patients with high AG metabolic acidosis (HR:0.88, *p* = 0.045) as shown in Table 5.

**Table 4:** Predictors of the use of sodium bicarbonate infusion at each time point.

	Odds ratio	Lower 95% CI	Upper 95% CI	*p* value
Gender (male as reference)	0.90	0.75	1.08	0.244
Age with 10 years increase	0.99	0.93	1.05	0.721
Mechanical ventilation	1.04	0.84	1.30	0.699
Fluid input with 1,000 increase	1.03	0.99	1.06	0.122
Urine output with 1,000 increase	1.05	0.96	1.14	0.294
pH with 0.1 increase	0.53	0.45	0.61	<0.001
Vasopressor	0.74	0.60	0.92	0.006
Anion gap with 5 increase	0.91	0.84	0.99	0.022
Lactate with 1 increase	1.05	1.02	1.08	0.002
BC (mmol/l) with 5 increase	0.39	0.34	0.44	<0.001
PaCO2 (mmHg) with 10 increase	1.08	0.96	1.22	0.208
Bun with 5 increase	1.01	0.99	1.03	0.298
Cre with 0.1 increase	1.00	0.99	1.00	0.225

**Table 5:** Results of marginal structural Cox model in overall population and subgroups.

Groups	HR	Lower.95	Upper.95	*p*
Overall population	0.92	0.82	1.04	0.179
AKI stage 1	0.77	0.55	1.08	0.126
AKI stage 2	1.10	0.97	1.24	0.131
AKI stage 3	0.88	0.74	1.03	0.106
AG>18	0.86	0.74	1.00	0.045

By considering 48 h SB volumes, the results showed that receiving 800–1,600 mL was associated with reduced risk of mortality as compared with the non-SB group (HR 0.56; 95% CI 0.31–0.99; p = 0.045). Beneficial effect was not observed in the small (dose <400 mL) and large volume (dose >1,600 mL) groups (Table 6).

**Table 6:** Dose–response relationship between SB volume and survival benefits in AKI patients with metabolic acidosis.

	HR	Lower.95	Upper.95	*p*
48 h SB volume (Non-SB group as reference)^*^				
SB < 400 mL	1.28	0.97	1.69	0.079
400–800 mL	0.93	0.68	1.27	0.656
800 to 1,600 mL	0.56	0.31	0.99	0.045
>1,600 mL	0.52	0.13	2.11	0.363

## Discussion

The present study focused on the effect of SB infusion on hospital mortality in AKI patients with metabolic acidosis. In our study, SB infusion was verified to not benefit all-stage AKI patients. But the improving hospital mortality of the AKI patients with high AG metabolic acidosis was associated with SB infusion; Previous studies have confirmed that high AG is an important predictor of all-cause death (18, 19), and the rate of patients with elevated AG requiring admission to the ICU and the mortality rate were significantly higher (20). Another study suggested that the risk of death of AKI patients within 28 days during hospitalization in the ICU was increased when AG ≥14 mmol/L (21).

The metabolic acidoses are readily subdivided into those with a large AG (HAGMA) and those with a normal AG (by definition the hyperchloremic metabolic acidoses). When HAGMA exists, the reciprocal relationship between the amount of increase of [AG] and decrease in [HCO3−] (designated as the Δ [AG]/Δ[HCO3−] can be a very helpful indicator of certain forms of mixed acid–base disorders (22)).

When HAGMA occurs, the ratio of Δ[AG] to Δ[HCO3-] is disrupted and the acid–base balance of the body is impaired. Infusion of SB provided a weak base and was able to correct the acidosis by adding bicarbonate base. The results of this study further confirm that SB infusion improves hospital mortality in AKI patients with high AG metabolic acidosis.

This study used PSM and MSCM and considered the effect of baseline and time-varying confounders. PH, PaCO2, lactate and BC were examined as time-varying factors. SB infusion and pH, PaCO2, lactate and BC are an interacting and dynamically changing relationship throughout the course of medical care, and the use of MSCM, can effectively minimize the effects of these confounding factors. Previous studies have confirmed that the MSCM model can be used for the observation and analysis of time variables and outcomes (23, 24).

In our study, SB infusion was verified to not benefit all-stage AKI patients. But the improving hospital mortality of the AKI patients with high AG metabolic acidosis was associated with SB infusion. This study also has some limitations, as an observational single-center study that retrospectively reviewed electronic health record (EHR) data, the potential for residual confounding by uncaptured variables in the EHR and the generalizability of the findings to other institutions require further investigation. Second, we used PSM and MSCM to balance important confounders, but residual confounders could not be completely excluded. Finally, we hope to explore this in some prospective trials and conduct further randomized controlled trials to confirm the results of this study.

## Conclusion

In conclusion, we found that the use of SB infusion in AKI patients complicated with metabolic acidosis has little association on hospital mortality. At the same time, we also found that timely use of SB for AKI patients with high AG acidosis significantly correlated with improving hospital mortality. Further larger randomized controlled trials are needed to confirm these results.

## Data availability statement

The raw data supporting the conclusions of this article will be made available by the authors, without undue reservation.

## Ethics statement

The studies involving humans were approved by Massachusetts Institute of Technology (Cambridge, MA) and Beth Israel Deaconess Medical Center (Boston, MA). The studies were conducted in accordance with the local legislation and institutional requirements. Written informed consent for participation was not required from the participants or the participants' legal guardians/next of kin in accordance with the national legislation and institutional requirements.

## Author contributions

YW: Data curation, Methodology, Writing – original draft, Writing – review & editing. LC: Data curation, Writing – review & editing. GG: Data curation, Writing – review & editing. YG: Methodology, Writing – review & editing. HG: Supervision, Writing – review & editing.
